# Crystal structure of ethyl (1*RS*,6*SR*)-4-(2-methyl-1*H*-imidazol-4-yl)-2-oxo-6-(2,3,5-tri­chloro­phen­yl)cyclo­hex-3-ene-1-carboxyl­ate

**DOI:** 10.1107/S2056989015023245

**Published:** 2016-01-01

**Authors:** Billava J. Mohan, Balladka K. Sarojini, Hemmige S. Yathirajan, Ravindranath Rathore, Christopher Glidewell

**Affiliations:** a Department of Chemistry, P.A. College of Engineering, Mangaluru 574 153, India; b Department of Studies in Industrial Chemistry, Mangalore University, Mangalagangothri 574 199, India; c Department of Studies in Chemistry, University of Mysore, Manasagangotri, Mysuru 570 006, India; d Department of Biotechnology, Dayananda Sagar College of Engineering, Bengaluru 560 078, India; e School of Chemistry, University of St Andrews, Fife KY16 9ST, Scotland

**Keywords:** crystal structure, cyclo­condensation reaction, mol­ecular stereochemistry, mol­ecular conformation, hydrogen bonding

## Abstract

The title compound, C_19_H_17_Cl_3_N_2_O_3_, has been prepared in a cyclo­condensation reaction between 2,3,5-tri­chloro­benzaldehye and 4-acetyl-2-methyl-1*H*-imidazole. The cyclo­hexenone ring adopts an envelope conformation with the C atom substituted by the tri­chloro­phenyl ring as the flap. The mutually *trans* ester and aryl substituents both occupy equatorial sites. In the crystal, a combination of N—H⋯O and C—H⋯N hydrogen bonds links the mol­ecules into ribbons of edge-fused centrosymmetric rings, which enclose *R*_2_^2^(14) and *R*_4_^4^(16) alternate ring motifs, propagating along the *b*-axis direction.

## Abstract 2

The cyclo­hexenone ring in the title compound adopts an envelope conformation and in the crystal, mol­ecules are linked by N—H⋯O and C—H⋯N hydrogen bonds, forming ribbons of edge-fused rings propagating along [010].

## Chemical context   

**Figure 1:**
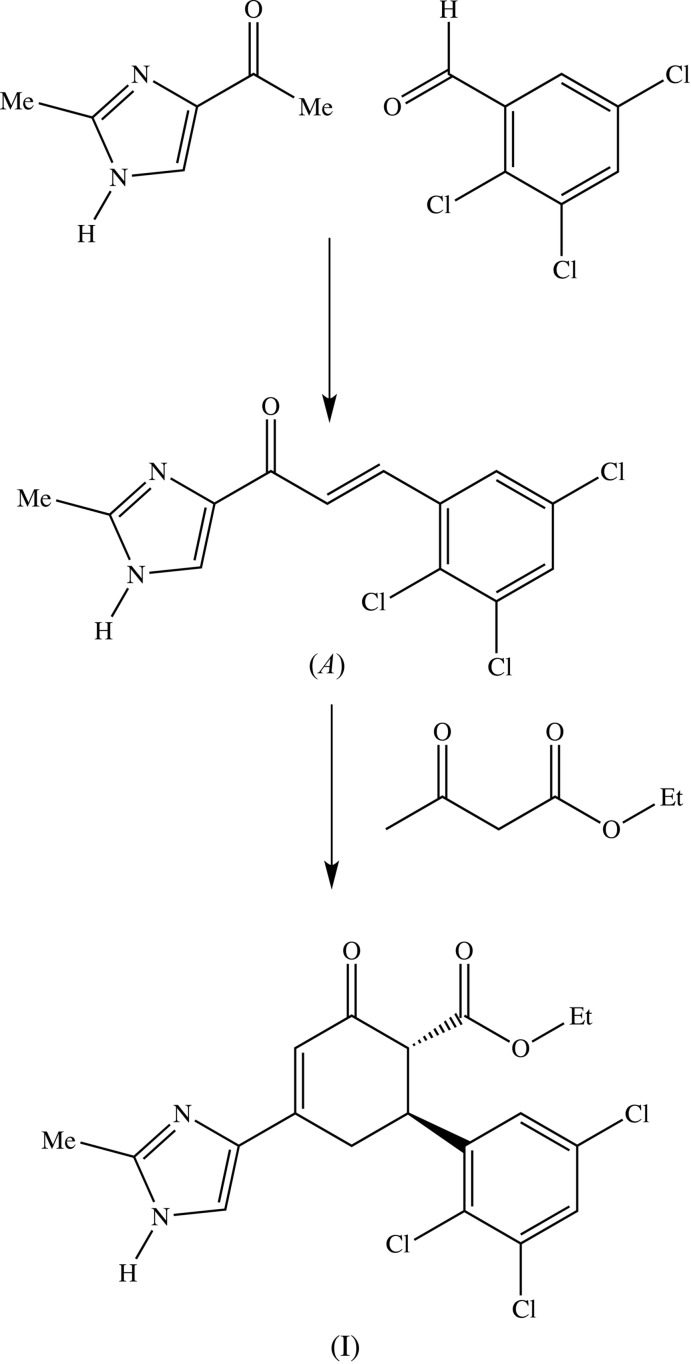
The synthesis of the title compound (I).

We have recently reported (Salian *et al.*, 2015 ▸) a simple and versatile synthesis of substituted 1,1′:3′1′′-terphenyls based upon the two-electron oxidation of substituted cyclo­hex-2-en-1-ones, themselves readily synthesized in reactions between 1,3-di­aryl­prop-2-en-1-ones (chalcones) and compounds containing activated methyl­ene units. This method points to a similar routes to substituted bi­phenyls carrying a wide range of substituents, including heterocyclic units. To this end, we have now synthesized the title compound (I) as a key inter­mediate in this proposed pathway. It was prepared by reaction of ethyl 3-oxo­butano­ate with the chalcone inter­mediate (*A*) (Fig. 1 ▸), which was itself prepared by base-catalysed condensation between 2,3,5-tri­chloro­benzaldehye and 4-acetyl-2-methyl-1*H*-imidazole. The conversion of the inter­mediate (*A*) to the final product (I) is a two-step, but one-pot, process involving both Michael addition and a condensation reaction.
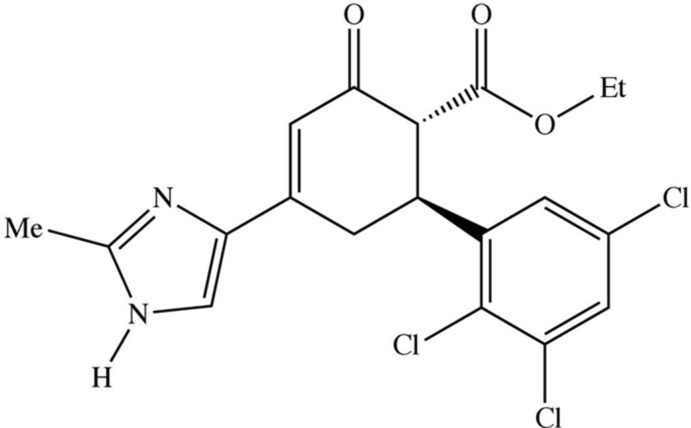


## Structural commentary   

**Figure 2:**
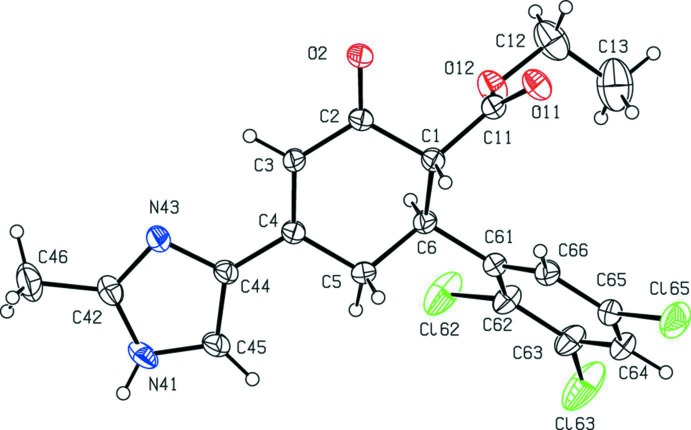
The mol­ecular structure of the (1*R*,6*S*) enanti­omer of compound (I), showing the atom-labelling scheme. Displacement ellipsoids are drawn at the 30% probability level.

The mol­ecule of compound (I) contains two stereogenic centres at atoms C1 and C6 (Fig. 2 ▸). The reference mol­ecule was selected as one having the *R*-configuration at atom C1 and in this mol­ecule atom C6 has the *S*-configuration; the centrosymmetric space group confirms that the compound has crystallized as a racemic mixture of the (1*R*,6*S*) and (1*S*,6*R*) diastereoisomers.

The central cyclo­hexenone ring (C1–C6), has puckering parameters of *Q* = 0.497 (3) Å, θ = 124.1 (3)° and φ = 123.6 (3)°, indicating an almost ideal envelope conformation with atom C6 as the flap. The maximum deviation from the mean plane through atoms (C1–C5) is 0.023 (2) Å for atom C4, with an r.m.s. deviation of 0.0144 Å, and with the flap atom C6 displaced by 0.684 (3) Å.

The ester and aryl substituents at atoms C1 and C6, respectively, are *trans* to one another and both occupy equatorial sites (Fig. 2 ▸). The dihedral angle between the mean plane through atoms (C1–C5) and the adjacent imidazole ring is only 2.18 (16)° but, despite this, the bond lengths in the imidazolyl-cyclohexenone portion of the molecule, atoms (N41,C45,C44,C4,C3,C2,O2), provide no evidence for delocal­ization of the lone pair from the planar atom N41 through the vinylogous amide fragment onto atom O2. In contrast, the dihedral angle between the mean plane through atoms (C1–C5) and the carboxyl group (C11/O11/O12) is 89.0 (3)°.

## Supra­molecular inter­actions   

**Figure 3:**
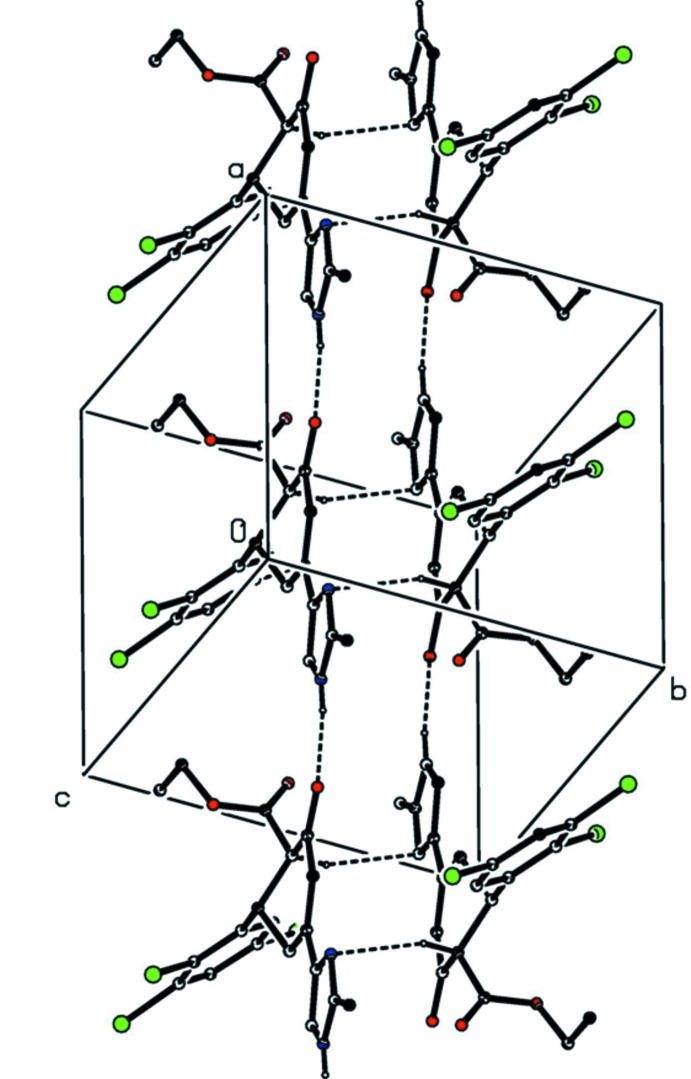
A partial view of the crystal packing of compound (I), showing the formation of a ribbon of edge-fused hydrogen-bonded 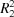
(14) and 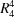
(16) rings running parallel to the [100] direction (see Table 1 ▸). Hydrogen bonds are shown as dashed lines and, for the sake of clarity, the H atoms not involved in the motifs shown have been omitted.

In the crystal of compound (I), mol­ecules related by translation along [100] are linked by nearly linear N—H⋯O hydrogen bonds (Table 1 ▸ and Fig. 3 ▸), forming *C*(8) chains, and inversion-related pairs of such chains are linked by C—H⋯N hydrogen bonds, forming ribbons or mol­ecular ladders of edge-fused centrosymmetric rings, in which 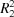
(14) rings centred at (*n* + 1/2, 1/2, 1/2) alternate with 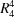
(16) rings centred at (*n*, 1/2, 1/2); where *n* represents an integer in each case (Fig. 3 ▸). There are no direction-specific inter­actions between adjacent ribbons.

## Database survey   

The structures of a number of analogues of compound (I), usually carrying aryl substituents on atoms C4 and C6, have been reported in recent years (Fischer *et al.*, 2008 ▸; Fun *et al.*, 2008 ▸, 2012 ▸; Dutkiewicz *et al.*, 2011*a* ▸,*b* ▸,*c* ▸; Kant *et al.*, 2012 ▸; Salian *et al.*, 2015 ▸). Without exception, these compounds all crystallize as racemic mixtures of the (1*R*,6*S*) and (1*S*,6*R*) forms, with mutually *trans* substituents at the sites corres­ponding to atoms C1 and C6 in compound (I), although in quite a number of these reports, the stereochemistry is not mentioned at all. The consistency of the stereochemistry indicates that the first step in the reaction between the chalcone and ester reagents is condensation between the chalcone and the acyl group of the ester component, followed by the Michael addition step, whose transition state is organized to minimize steric repulsions, leading to the mutually *trans* disposition of the substituents at sites C1 and C6. Of particular inter­est is the structure of methyl (1*RS*,6*SR*)-4-(4-chlorophen­yl)-6-[4-(propan-2-yl)phen­yl]-2-oxo­cyclo­hex-3-ene-1-carb­oxyl­ate, which exhibits enanti­omeric disorder where the reference site contains both (1*R*,6S) and (1*S*,6*R*) forms with occupancies of 0.923 (3) and 0.077 (3), respectively (Salian *et al.*, 2015 ▸), There appears to be no evidence for such disorder in the structure reported earlier nor, indeed, in the structure of compound (I) reported here.

## Synthesis and crystallization   

The synthesis of the title compound is illustrated in Fig. 1 ▸. For the synthesis of 1-(2-methyl-1*H*-imidazol-4-yl)-3-(2,3,5-tri­chloro­phen­yl)prop-2-en-1-one (*A*), aqueous sodium hydrox­ide solution (10% *w*/*v*, 30 cm^3^) was added to a mixture of 2,3,5- tri­chloro­benzaldehyde (0.02 mol) and 4-acetyl-2-methyl-1*H*-imidazole (0.02 mol), and the mixture was stirred at 275 K for 3 h. The resulting solid product was collected by filtration and recrystallized from ethanol. For the synthesis of the title compound, (I), a mixture of compound *A* (3.15 g, 0.01 mol) and ethyl 3-oxo­butano­ate (1.30 g, 0.01 mol) in methanol (30 cm^3^) containing aqueous sodium hydroxide (10% *w*/*v*, 0.8 cm^3^) was heated under reflux for 10 h. The reaction mixture was then cooled to ambient temperature and the resulting solid product (I) was collected by filtration. Crystals suitable for single-crystal X-ray diffraction were grown by slow evaporation, at ambient temperature and in the presence of air, of a solution in methanol.

## Refinement   

Crystal data, data collection and structure refinement details are summarized in Table 2 ▸. All the H atoms were located in difference-Fourier maps. For the H atom bonded to atom N41, the atomic coordinates were refined with *U*_iso_(H) = 1.2*U*_eq_(N), giving an N—H distance of 0.79 (3) Å. The C-bound H atoms were subsequently treated as riding atoms in geometrically idealized positions: C—H distances 0.93–98 Å with *U*_iso_(H) = 1.5*U*_eq_(Cmeth­yl) and 1.2*U*_eq_(C) for other H atoms.

## Supplementary Material

Crystal structure: contains datablock(s) global, I. DOI: 10.1107/S2056989015023245/su5251sup1.cif

Structure factors: contains datablock(s) I. DOI: 10.1107/S2056989015023245/su5251Isup2.hkl

Supporting information file. DOI: 10.1107/S2056989015023245/su5251Isup3.cml

CCDC reference: 1440150

Additional supporting information:  crystallographic information; 3D view; checkCIF report

## Figures and Tables

**Table 1:** Hydrogen-bond geometry (Å, °)

*D*—H⋯*A*	*D*—H	H⋯*A*	*D*⋯*A*	*D*—H⋯*A*
N41—H41⋯O2^i^	0.79 (4)	2.10 (4)	2.878 (3)	167 (3)
C1—H1⋯N43^ii^	0.98	2.60	3.538 (4)	161

Symmetry codes: (i) 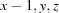; (ii) 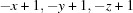.

**Table 2:** Experimental details

Crystal data	
Chemical formula	C_19_H_17_Cl_3_N_2_O_3_
*M*_r_	427.70
Crystal system, space group	Triclinic, *P* 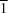
Temperature (K)	295
*a*, *b*, *c* (Å)	9.753 (5), 10.029 (6), 11.099 (5)
α, β, γ (°)	106.281 (4), 96.420 (5), 104.913 (5)
*V* (Å^3^)	987.0 (9)
*Z*	2
Radiation type	Mo *K*α
μ (mm^−1^)	0.49
Crystal size (mm)	0.26 × 0.21 × 0.18

Data collection	
Diffractometer	Bruker APEXII area detector
Absorption correction	Multi-scan (*SADABS*; Sheldrick, 2003 ▸)
*T*_min_, *T*_max_	0.789, 0.916
No. of measured, independent and observed [*I* > 2σ(*I*)] reflections	18900, 4534, 3178
*R*_int_	0.026
(sin θ/λ)_max_ (Å^−1^)	0.651

Refinement	
*R*[*F* ^2^ > 2σ(*F* ^2^)], *wR*(*F* ^2^), *S*	0.050, 0.146, 1.03
No. of reflections	4534
No. of parameters	249
H-atom treatment	H atoms treated by a mixture of independent and constrained refinement
Δρ_max_, Δρ_min_ (e Å^−3^)	0.58, −0.54

Computer programs: *APEX2* and *SAINT-Plus* (Bruker, 2012 ▸), *SHELXS97* (Sheldrick, 2008 ▸), *SHELXL2014* (Sheldrick, 2015 ▸) and *PLATON* (Spek, 2009 ▸).

## supplementary crystallographic information

### Crystal data

**Table:**

C_19_H_17_Cl_3_N_2_O_3_	*Z* = 2
*M**_r_* = 427.70	*F*(000) = 440
Triclinic, *P*1	*D*_x_ = 1.439 Mg m^−^^3^
*a* = 9.753 (5) Å	Mo *K*α radiation, λ = 0.71073 Å
*b* = 10.029 (6) Å	Cell parameters from 5020 reflections
*c* = 11.099 (5) Å	θ = 2.7–28.7°
α = 106.281 (4)°	µ = 0.49 mm^−^^1^
β = 96.420 (5)°	*T* = 295 K
γ = 104.913 (5)°	Block, colourless
*V* = 987.0 (9) Å^3^	0.26 × 0.21 × 0.18 mm

### Data collection

**Table:**

Bruker APEXII area-detector diffractometer	4534 independent reflections
Radiation source: fine-focus sealed tube	3178 reflections with *I* > 2σ(*I*)
Graphite monochromator	*R*_int_ = 0.026
φ and ω scans	θ_max_ = 27.6°, θ_min_ = 2.2°
Absorption correction: multi-scan (*SADABS*; Sheldrick, 2003)	*h* = −12→12
*T*_min_ = 0.789, *T*_max_ = 0.916	*k* = −12→13
18900 measured reflections	*l* = −14→14

### Refinement

**Table:**

Refinement on *F*^2^	0 restraints
Least-squares matrix: full	Hydrogen site location: mixed
*R*[*F*^2^ > 2σ(*F*^2^)] = 0.050	H atoms treated by a mixture of independent and constrained refinement
*wR*(*F*^2^) = 0.146	*w* = 1/[σ^2^(*F*_o_^2^) + (0.0533*P*)^2^ + 0.9642*P*] where *P* = (*F*_o_^2^ + 2*F*_c_^2^)/3
*S* = 1.03	(Δ/σ)_max_ < 0.001
4534 reflections	Δρ_max_ = 0.58 e Å^−^^3^
249 parameters	Δρ_min_ = −0.54 e Å^−^^3^

### Special details

**Table:**

Geometry. All e.s.d.'s (except the e.s.d. in the dihedral angle between two l.s. planes) are estimated using the full covariance matrix. The cell e.s.d.'s are taken into account individually in the estimation of e.s.d.'s in distances, angles and torsion angles; correlations between e.s.d.'s in cell parameters are only used when they are defined by crystal symmetry. An approximate (isotropic) treatment of cell e.s.d.'s is used for estimating e.s.d.'s involving l.s. planes.

### Fractional atomic coordinates and isotropic or equivalent isotropic displacement parameters (Å^2^)

**Table:**

	*x*	*y*	*z*	*U*_iso_*/*U*_eq_	
C1	0.6401 (2)	0.3358 (2)	0.5952 (2)	0.0332 (5)	
H1	0.6710	0.4417	0.6366	0.040*	
C2	0.6017 (2)	0.3076 (3)	0.4517 (2)	0.0343 (5)	
O2	0.69386 (18)	0.3027 (2)	0.38603 (16)	0.0473 (4)	
C3	0.4555 (2)	0.2959 (3)	0.3987 (2)	0.0354 (5)	
H3	0.4295	0.2757	0.3108	0.042*	
C4	0.3547 (2)	0.3129 (2)	0.4705 (2)	0.0331 (5)	
C5	0.3877 (2)	0.3355 (3)	0.6115 (2)	0.0370 (5)	
H5A	0.3007	0.2896	0.6373	0.044*	
H5B	0.4171	0.4391	0.6583	0.044*	
C6	0.5070 (2)	0.2725 (3)	0.6461 (2)	0.0336 (5)	
H6	0.4723	0.1671	0.6021	0.040*	
C11	0.7669 (3)	0.2835 (3)	0.6267 (2)	0.0378 (5)	
O11	0.88835 (19)	0.3608 (2)	0.66651 (19)	0.0516 (5)	
O12	0.7266 (2)	0.1410 (2)	0.6038 (2)	0.0547 (5)	
C12	0.8381 (4)	0.0789 (4)	0.6394 (4)	0.0731 (10)	
H12A	0.9302	0.1321	0.6260	0.088*	
H12B	0.8140	−0.0219	0.5857	0.088*	
C13	0.8502 (6)	0.0866 (5)	0.7739 (5)	0.1074 (16)	
H13A	0.7588	0.0341	0.7870	0.161*	
H13B	0.8767	0.1867	0.8270	0.161*	
H13C	0.9229	0.0441	0.7961	0.161*	
N41	−0.0094 (2)	0.3117 (3)	0.3823 (2)	0.0491 (6)	
H41	−0.086 (4)	0.321 (3)	0.393 (3)	0.059*	
C42	0.0373 (3)	0.2879 (3)	0.2703 (3)	0.0452 (6)	
N43	0.1716 (2)	0.2869 (2)	0.28599 (19)	0.0404 (5)	
C44	0.2128 (2)	0.3098 (3)	0.4154 (2)	0.0344 (5)	
C45	0.1003 (3)	0.3263 (3)	0.4751 (3)	0.0435 (6)	
H45	0.0996	0.3440	0.5618	0.052*	
C46	−0.0557 (3)	0.2675 (4)	0.1471 (3)	0.0726 (10)	
H46A	−0.0195	0.2161	0.0773	0.109*	
H46B	−0.0548	0.3609	0.1399	0.109*	
H46C	−0.1530	0.2122	0.1442	0.109*	
C61	0.5373 (2)	0.2972 (3)	0.7887 (2)	0.0351 (5)	
C62	0.4533 (3)	0.2008 (3)	0.8401 (2)	0.0453 (6)	
Cl62	0.31485 (11)	0.05093 (9)	0.74148 (7)	0.0798 (3)	
C63	0.4784 (3)	0.2242 (3)	0.9712 (3)	0.0538 (7)	
Cl63	0.37058 (14)	0.10827 (13)	1.03524 (9)	0.1058 (5)	
C64	0.5884 (3)	0.3421 (3)	1.0529 (2)	0.0508 (7)	
H64	0.6071	0.3565	1.1404	0.061*	
C65	0.6695 (3)	0.4379 (3)	1.0018 (2)	0.0454 (6)	
Cl65	0.80666 (9)	0.58707 (10)	1.10331 (7)	0.0736 (3)	
C66	0.6450 (3)	0.4174 (3)	0.8725 (2)	0.0401 (5)	
H66	0.7012	0.4848	0.8410	0.048*	

### Atomic displacement parameters (Å^2^)

**Table:**

	*U*^11^	*U*^22^	*U*^33^	*U*^12^	*U*^13^	*U*^23^
C1	0.0278 (10)	0.0376 (12)	0.0331 (11)	0.0076 (9)	0.0063 (9)	0.0118 (9)
C2	0.0283 (11)	0.0410 (12)	0.0354 (12)	0.0108 (9)	0.0097 (9)	0.0134 (10)
O2	0.0297 (8)	0.0772 (13)	0.0393 (9)	0.0183 (8)	0.0135 (7)	0.0209 (9)
C3	0.0285 (11)	0.0505 (14)	0.0285 (11)	0.0115 (10)	0.0070 (8)	0.0144 (10)
C4	0.0287 (11)	0.0377 (12)	0.0328 (11)	0.0086 (9)	0.0073 (9)	0.0121 (9)
C5	0.0330 (12)	0.0501 (14)	0.0313 (11)	0.0154 (10)	0.0118 (9)	0.0140 (10)
C6	0.0309 (11)	0.0405 (12)	0.0293 (11)	0.0088 (9)	0.0077 (9)	0.0123 (9)
C11	0.0357 (12)	0.0470 (14)	0.0335 (12)	0.0142 (10)	0.0089 (9)	0.0150 (10)
O11	0.0310 (9)	0.0553 (11)	0.0652 (12)	0.0095 (8)	0.0048 (8)	0.0193 (9)
O12	0.0439 (10)	0.0450 (11)	0.0700 (13)	0.0124 (8)	−0.0010 (9)	0.0161 (9)
C12	0.063 (2)	0.0548 (19)	0.107 (3)	0.0243 (16)	0.0036 (19)	0.0332 (19)
C13	0.121 (4)	0.098 (3)	0.113 (4)	0.033 (3)	−0.008 (3)	0.061 (3)
N41	0.0257 (10)	0.0629 (15)	0.0679 (15)	0.0175 (10)	0.0148 (10)	0.0295 (12)
C42	0.0323 (12)	0.0508 (15)	0.0526 (15)	0.0106 (11)	0.0030 (11)	0.0208 (12)
N43	0.0308 (10)	0.0521 (12)	0.0397 (11)	0.0137 (9)	0.0050 (8)	0.0166 (9)
C44	0.0268 (10)	0.0400 (12)	0.0375 (12)	0.0091 (9)	0.0082 (9)	0.0144 (10)
C45	0.0312 (12)	0.0572 (16)	0.0476 (14)	0.0154 (11)	0.0142 (10)	0.0209 (12)
C46	0.0450 (17)	0.099 (3)	0.070 (2)	0.0196 (17)	−0.0092 (15)	0.0305 (19)
C61	0.0336 (11)	0.0425 (13)	0.0310 (11)	0.0139 (9)	0.0075 (9)	0.0120 (10)
C62	0.0542 (15)	0.0442 (14)	0.0324 (12)	0.0069 (11)	0.0110 (11)	0.0109 (10)
Cl62	0.0963 (7)	0.0659 (5)	0.0445 (4)	−0.0258 (4)	0.0122 (4)	0.0142 (4)
C63	0.0669 (18)	0.0590 (17)	0.0371 (14)	0.0121 (14)	0.0189 (13)	0.0212 (13)
Cl63	0.1377 (10)	0.1048 (8)	0.0488 (5)	−0.0215 (7)	0.0276 (5)	0.0344 (5)
C64	0.0556 (16)	0.0693 (18)	0.0284 (12)	0.0232 (14)	0.0089 (11)	0.0132 (12)
C65	0.0390 (13)	0.0565 (16)	0.0340 (12)	0.0166 (11)	0.0019 (10)	0.0044 (11)
Cl65	0.0584 (5)	0.0874 (6)	0.0435 (4)	−0.0011 (4)	−0.0029 (3)	−0.0008 (4)
C66	0.0354 (12)	0.0484 (14)	0.0353 (12)	0.0111 (10)	0.0077 (10)	0.0130 (10)

### Geometric parameters (Å, º)

**Table:**

C1—C11	1.506 (3)	C13—H13C	0.9600
C1—C2	1.522 (3)	N41—C45	1.348 (3)
C1—C6	1.532 (3)	N41—C42	1.351 (4)
C1—H1	0.9800	N41—H41	0.79 (3)
C2—O2	1.221 (3)	C42—N43	1.306 (3)
C2—C3	1.443 (3)	C42—C46	1.485 (4)
C3—C4	1.347 (3)	N43—C44	1.384 (3)
C3—H3	0.9300	C44—C45	1.365 (3)
C4—C44	1.438 (3)	C45—H45	0.9300
C4—C5	1.502 (3)	C46—H46A	0.9600
C5—C6	1.521 (3)	C46—H46B	0.9600
C5—H5A	0.9700	C46—H46C	0.9600
C5—H5B	0.9700	C61—C66	1.387 (3)
C6—C61	1.514 (3)	C61—C62	1.391 (3)
C6—H6	0.9800	C62—C63	1.390 (4)
C11—O11	1.191 (3)	C62—Cl62	1.727 (3)
C11—O12	1.322 (3)	C63—C64	1.378 (4)
O12—C12	1.454 (4)	C63—Cl63	1.722 (3)
C12—C13	1.463 (6)	C64—C65	1.373 (4)
C12—H12A	0.9700	C64—H64	0.9300
C12—H12B	0.9700	C65—C66	1.376 (3)
C13—H13A	0.9600	C65—Cl65	1.727 (3)
C13—H13B	0.9600	C66—H66	0.9300
			
C11—C1—C2	110.53 (18)	C12—C13—H13C	109.5
C11—C1—C6	113.77 (19)	H13A—C13—H13C	109.5
C2—C1—C6	110.99 (18)	H13B—C13—H13C	109.5
C11—C1—H1	107.1	C45—N41—C42	108.0 (2)
C2—C1—H1	107.1	C45—N41—H41	125 (2)
C6—C1—H1	107.1	C42—N41—H41	127 (2)
O2—C2—C3	121.8 (2)	N43—C42—N41	111.5 (2)
O2—C2—C1	120.7 (2)	N43—C42—C46	125.8 (3)
C3—C2—C1	117.43 (18)	N41—C42—C46	122.7 (3)
C4—C3—C2	123.1 (2)	C42—N43—C44	105.2 (2)
C4—C3—H3	118.4	C45—C44—N43	109.6 (2)
C2—C3—H3	118.4	C45—C44—C4	128.4 (2)
C3—C4—C44	121.5 (2)	N43—C44—C4	121.97 (19)
C3—C4—C5	120.7 (2)	N41—C45—C44	105.8 (2)
C44—C4—C5	117.83 (19)	N41—C45—H45	127.1
C4—C5—C6	111.86 (18)	C44—C45—H45	127.1
C4—C5—H5A	109.2	C42—C46—H46A	109.5
C6—C5—H5A	109.2	C42—C46—H46B	109.5
C4—C5—H5B	109.2	H46A—C46—H46B	109.5
C6—C5—H5B	109.2	C42—C46—H46C	109.5
H5A—C5—H5B	107.9	H46A—C46—H46C	109.5
C61—C6—C5	110.43 (18)	H46B—C46—H46C	109.5
C61—C6—C1	113.84 (18)	C66—C61—C62	117.8 (2)
C5—C6—C1	109.12 (19)	C66—C61—C6	121.8 (2)
C61—C6—H6	107.7	C62—C61—C6	120.3 (2)
C5—C6—H6	107.7	C63—C62—C61	120.7 (2)
C1—C6—H6	107.7	C63—C62—Cl62	119.0 (2)
O11—C11—O12	124.3 (2)	C61—C62—Cl62	120.26 (19)
O11—C11—C1	124.2 (2)	C64—C63—C62	120.7 (2)
O12—C11—C1	111.6 (2)	C64—C63—Cl63	118.6 (2)
C11—O12—C12	116.6 (2)	C62—C63—Cl63	120.7 (2)
O12—C12—C13	110.1 (3)	C65—C64—C63	118.3 (2)
O12—C12—H12A	109.6	C65—C64—H64	120.9
C13—C12—H12A	109.6	C63—C64—H64	120.9
O12—C12—H12B	109.6	C64—C65—C66	121.7 (2)
C13—C12—H12B	109.6	C64—C65—Cl65	118.8 (2)
H12A—C12—H12B	108.1	C66—C65—Cl65	119.5 (2)
C12—C13—H13A	109.5	C65—C66—C61	120.7 (2)
C12—C13—H13B	109.5	C65—C66—H66	119.7
H13A—C13—H13B	109.5	C61—C66—H66	119.7
			
C11—C1—C2—O2	−27.7 (3)	C42—N43—C44—C4	178.7 (2)
C6—C1—C2—O2	−154.8 (2)	C3—C4—C44—C45	−179.5 (3)
C11—C1—C2—C3	156.0 (2)	C5—C4—C44—C45	0.7 (4)
C6—C1—C2—C3	28.9 (3)	C3—C4—C44—N43	1.1 (4)
O2—C2—C3—C4	−174.3 (2)	C5—C4—C44—N43	−178.8 (2)
C1—C2—C3—C4	1.9 (3)	C42—N41—C45—C44	−0.3 (3)
C2—C3—C4—C44	175.9 (2)	N43—C44—C45—N41	0.8 (3)
C2—C3—C4—C5	−4.2 (4)	C4—C44—C45—N41	−178.8 (2)
C3—C4—C5—C6	−24.9 (3)	C5—C6—C61—C66	−93.7 (3)
C44—C4—C5—C6	154.9 (2)	C1—C6—C61—C66	29.5 (3)
C4—C5—C6—C61	−179.98 (19)	C5—C6—C61—C62	84.2 (3)
C4—C5—C6—C1	54.2 (3)	C1—C6—C61—C62	−152.7 (2)
C11—C1—C6—C61	54.8 (3)	C66—C61—C62—C63	−0.7 (4)
C2—C1—C6—C61	−179.81 (19)	C6—C61—C62—C63	−178.6 (2)
C11—C1—C6—C5	178.64 (19)	C66—C61—C62—Cl62	178.65 (19)
C2—C1—C6—C5	−56.0 (2)	C6—C61—C62—Cl62	0.7 (3)
C2—C1—C11—O11	99.8 (3)	C61—C62—C63—C64	−1.0 (4)
C6—C1—C11—O11	−134.6 (2)	Cl62—C62—C63—C64	179.6 (2)
C2—C1—C11—O12	−79.8 (2)	C61—C62—C63—Cl63	177.8 (2)
C6—C1—C11—O12	45.8 (3)	Cl62—C62—C63—Cl63	−1.5 (4)
O11—C11—O12—C12	4.8 (4)	C62—C63—C64—C65	1.9 (4)
C1—C11—O12—C12	−175.6 (2)	Cl63—C63—C64—C65	−177.0 (2)
C11—O12—C12—C13	86.1 (4)	C63—C64—C65—C66	−1.1 (4)
C45—N41—C42—N43	−0.2 (3)	C63—C64—C65—Cl65	179.4 (2)
C45—N41—C42—C46	−179.7 (3)	C64—C65—C66—C61	−0.7 (4)
N41—C42—N43—C44	0.7 (3)	Cl65—C65—C66—C61	178.85 (19)
C46—C42—N43—C44	−179.9 (3)	C62—C61—C66—C65	1.5 (4)
C42—N43—C44—C45	−0.9 (3)	C6—C61—C66—C65	179.4 (2)

### Hydrogen-bond geometry (Å, º)

**Table:**

*D*—H···*A*	*D*—H	H···*A*	*D*···*A*	*D*—H···*A*
N41—H41···O2^i^	0.79 (4)	2.10 (4)	2.878 (3)	167 (3)
C1—H1···N43^ii^	0.98	2.60	3.538 (4)	161

Symmetry codes: (i) *x*−1, *y*, *z*; (ii) −*x*+1, −*y*+1, −*z*+1.
